# Characterization of subacute and convalescent fibrotic burden in the remote myocardium after acute infarction provides strong and incremental prediction of changes in left and right functions and final infarct size, incremental to knowledge of the subacute infarct size

**DOI:** 10.1186/1532-429X-14-S1-P18

**Published:** 2012-02-01

**Authors:** Damien Mandry, Evan Appelbaum, Bobby Heydari, Shuaib Abdullah, Sanjeev Francis, Heidi S Lumish, Brian B Ghoshhajra, Udo Hoffmann, Henry Gewirtz, Ron Blankstein, Yucheng Chen, Jiazuo H Feng, Karl-Philipp Kienle, Rob J van der Geest, Elliott Antman, Michael Jerosch-Herold, Raymond Kwong

**Affiliations:** 1 Cardiovascular Division, Department of Medicine, Brigham and Women's Hospital, Boston, MA, USA; 2 Cardiology Division, Massachusetts General Hospital, Boston, MA, USA; 3 Department of Radiology, Massachusetts General Hospital, Boston, MA, USA; 4 Leiden University Medical Center, Leiden, Netherlands, USA; 5 Department of Radiology, Brigham and Women's Hospital, Boston, MA, USA

## Summary

To test the hypothesis that fibrotic burden in remote myocardium quantified by CMR during early period of infarct healing is a strong determinant of the cardiac remodeling outcome.

## Background

After acute myocardial infarction (AMI), co-existing myocardial stunning, ischemia, and architectural alteration may yield variable patterns of ventricular remodeling with potential long-term prognostic implications. We hypothesize that CMR quantification of fibrotic content, based on R1 (1/T1) assessment, in non-infarct myocardium early after infarction and during the infarct healing phases may provide novel prediction of the final infarct size and alteration of ventricular functions during infarct healing.

## Methods

We quantified infarct size by late gadolinium enhancement (LGE), mean segmental fibrotic index (FI_MeanSeg_) and total LV fibrotic burden (FI_Total_), ventricular function and sizes during subacute (2-4 weeks) and chronic stages (6 months) of healing in 62 AMI patients (45 men, mean LVEF 55±8%). Infarct-healing period was defined as the time between the subacute and the chronic stages. For the purpose of quantifying FI_MeanSeg_ and FI_Total_, we serially quantified the ratio of myocardial to blood pool R1 changes (from before, and up to 30 minutes after contrast injection) to measure the expansion of the extracellular matrix as a marker of fibrosis. Three parallel short-axis slices were acquired for fibrotic quantification using a cine-IR sequence and slice-matched LGE imaging. Myocardial segments with any LGE were excluded from quantification of fibrotic indices. Serum hematocrit was used to estimate the plasma R1 from the measured blood R1.

## Results

From subacute to chronic stage, FI_MeanSeg_ in remote myocardium increased significantly (from 0.32±0.05 to 0.35±0.07, P=0.008). Severity of increased FI_MeanSeg_ during the infarct-healing period was correlated to the severity in worsening of LVEF (r=-0.27, P=0.03), trend worsening of LVESV (r=0.24, P=0.07), and significant worsening of RVESV (r=0.34, P=0.009). Linear regression demonstrated that FI_MeanSeg_ was one of the strongest predictors of final infarct mass (in grams) at the chronic stage. Specifically, both FI_MeanSeg_ at the subacute stage and its change during the infarct-healing period, demonstrated strong and independent association with the extent of the final infarct mass at the chronic stage, incremental to infarct mass measured at the subacute stage (P=0.004 and P=0.02, respectively, for improved model F-value).

## Conclusions

Quantification of non-infarcted fibrotic burden during subacute stage and its change during infarct-healing provides strong prediction of the severity of the LV and RV post-MI remodeling and of final infarct size at 6 months.

## Funding

National Heart Lung and Blood Institute, National Institutes of Health (RO1-HL091157).

Dr. Mandry’s salary is supported by the Societe Francaise de Radiologie (France).

